# First-Principles Study on Janus-Structured Sc_2_CX_2_/Sc_2_CY_2_ (X, Y = F, Cl, Br) Heterostructures for Solar Energy Conversion

**DOI:** 10.3390/molecules29122898

**Published:** 2024-06-18

**Authors:** Xin He, Yanan Wu, Jia Luo, Xianglin Dai, Jun Song, Yong Tang

**Affiliations:** 1 School of Energy Engineering, Huanghuai University, Zhumadian 463000, China; 20202086@huanghuai.edu.cn (X.H.); wuyanan@huanghuai.edu.cn (Y.W.); 10505182024@163.com (J.L.); 20202131@huanghuai.edu.cu (X.D.); songjunaa@163.com (J.S.); 2 Henan Key Laboratory of Smart Lighting, Huanghuai University, Zhumadian 463000, China

**Keywords:** Sc_2_CX_2_/Sc_2_CY_2_ (X, Y = F, Cl, Br) heterostructures, first-principles calculations, direct Z-scheme photocatalyst, photovoltaic applications

## Abstract

Two-dimensional van der Waals heterostructures have good application prospects in solar energy conversion due to their excellent optoelectronic performance. In this work, the electronic structures of Sc_2_CF_2_/Sc_2_CCl_2_, Sc_2_CF_2_/Sc_2_CBr_2_, and Sc_2_CCl_2_/Sc_2_CBr_2_ heterostructures, as well as their properties in photocatalysis and photovoltaics, have been comprehensively studied using the first-principles method. Firstly, both of the three thermodynamically and dynamically stable heterostructures are found to have type-II band alignment with band gap values of 0.58 eV, 0.78 eV, and 1.35 eV. Meanwhile, the photogenerated carriers in Sc_2_CF_2_/Sc_2_CCl_2_ and Sc_2_CF_2_/Sc_2_CBr_2_ heterostructures are predicated to follow the direct Z-scheme path, enabling their abilities for water splitting. As for the Sc_2_CCl_2_/Sc_2_CBr_2_ heterostructure, its photovoltaic conversion efficiency is estimated to be 20.78%. Significantly, the light absorption coefficients of Sc_2_CF_2_/Sc_2_CCl_2_, Sc_2_CF_2_/Sc_2_CBr_2_, and Sc_2_CCl_2_/Sc_2_CBr_2_ heterostructures are enhanced more than those of the corresponding monolayers. Moreover, biaxial strains have been observed to considerably tune the aforementioned properties of heterostructures. All the theoretical results presented in this work demonstrate the application potential of Sc_2_CX_2_/Sc_2_CY_2_ (X, Y = F, Cl, Br) heterostructures in photocatalysis and photovoltaics.

## 1. Introduction

With the depletion of traditional fossil fuels and the escalating global energy crisis, it is imperative and urgent to explore green and renewable energy sources. The use of semiconductor materials in applications such as photocatalysis or solar cells to convert abundant solar energy into clean power holds significant promise [1]. For instance, Fujishima and Honda were pioneers in demonstrating that TiO_2_ could serve as a photocatalyst for water splitting [2]. Nevertheless, the efficiency of TiO_2_ in converting solar energy to hydrogen is hindered by its wide band gap and high rate of carrier recombination. Chapin et al. were the first to create a solar cell using single-crystal silicon as the primary material. However, the photoelectric conversion efficiency (PCE) was disappointingly low, measuring only 6% [3]. As a result, the quest for suitable materials for photocatalysis and photovoltaics has been a prominent research area for a considerable period of time.

The discovery of graphene has sparked researchers’ interest in two-dimensional (2D) materials [4,5]. The 2D materials demonstrate amazing properties, including high carrier mobility, a semiconducting band gap, prominent catalytic activities, and abundant active sites. Therefore, they can be utilized in the fields of photocatalytic water splitting and photovoltaics. At present, many 2D materials have been synthesized experimentally or theoretically, such as transition metal carbides/nitrides (MXenes) [6], transition metal dichalcogenides (TMDCs) [7], hexagonal boron nitride (h-BN) [8], black phosphorus (BP) [9], and silicene [10]. However, 2D materials have a large band gap, poor light absorption capacity, and a high carrier recombination rate, thereby leading to low efficiency. Therefore, various strategic techniques such as doping [11], metal loading [12], and constructing heterostructures have been proposed. Among these strategies, constructing van der Waals (vdW) heterostructures with type-II band alignment has promising applications in the fields of photocatalytic water splitting and solar cells due to the lower exciton binding energy and enhanced optical absorbance compared to monolayers [13]. In type-II heterostructures, the photogenerated electron–hole pairs are separated onto different monolayers, which significantly reduces the carrier recombination rate. With the deepening of research, direct Z-scheme heterostructures can be designed by selecting two appropriate monolayer materials. In the Z-scheme heterostructure, photogenerated electrons and holes accumulate on the surfaces of distinct monolayers. The Z-scheme heterostructure not only possesses a strong redox ability to drive photocatalytic reactions but also provides active sites for spatially separated oxidation and reduction processes [14]. This mechanism significantly enhances the efficiency of water splitting in the heterostructure. According to previous research, the narrow band gap of the direct Z-scheme heterostructures can achieve a broader range of solar energy harvesting [15]. The Z-scheme heterostructures show great promise in photocatalytic water splitting, photocatalytic reduction of carbon dioxide, and environmental remediation [16,17]. In recent years, more and more Z-scheme heterostructures have been discovered and studied. Indeed, examples such as the WO_3_/Bi_2_MoO_6_ heterostructure [18], β-SnSe/HfS_2_ heterostructure [19], GaSe/ZrS_2_ heterostructure [20], MoSTe/g-GeC heterostructure [21], GeC/BSe heterostructure [22], and SnC/PtS_2_ heterostructure [23] all represent direct Z-scheme heterostructures.

On the other hand, MXenes have been widely explored in applications such as photocatalysts, solar cells, heavy-metal removal, battery anodes, and electromagnetic interference shielding. MXenes are produced from their corresponding MAX phases, where M represents an early transition metal, A represents a group of IIIA or IVA elements, and X represents a C or N atom [24]. MXenes have attracted increasing attention due to their excellent stability and large specific surface area. In the field of photocatalysis, heterostructures based on MXenes, such as Cs_2_AgBiBr_6_/Ti_3_C_2_T_x_ [25], Hf_2_CO_2_/WS_2_ [26], AsP/Sc_2_CO_2_ [27], and Sc_2_CF_2_/MoSSe [28], exhibit superior electronic properties. For the application of solar cells, Wen et al. demonstrated that the PCE of Hf_2_CO_2_/MoS_2_ and Zr_2_CO_2_/MoS_2_ heterostructures in solar cell applications was 19.75% and 17.13%, respectively [29]. The PCE of Ti_2_CO_2_/Zr_2_CO_2_ and Ti_2_CO_2_/Hf_2_CO_2_ heterostructures reaches 22.74% and 19.56%, respectively [30]. This indicates that MXenes have promising potential for applications as photovoltaic materials. Pure Sc_2_C exhibits metallic properties; however, after functionalization by F, Cl, and Br atoms, Sc_2_CF_2_, Sc_2_CCl_2_, and Sc_2_CBr_2_ exhibit semiconductor characteristics with band gaps of 1.85 eV, 1.70 eV, and 1.54 eV, respectively [31]. As a member of MXenes, Sc_2_CX_2_ (X = F, Cl, Br) exhibits kinetic and thermal stabilities, which have potential applications in photocatalytic water splitting and solar cells [32]. However, the Sc_2_CF_2_ monolayer cannot facilitate the oxygen evolution reaction (OER) because its valence band maximum (VBM) is higher than that of E_O2/H2O_. For Sc_2_CCl_2_ and Sc_2_CBr_2_ monolayers, the conduction band minimum (CBM) is lower than E_H+/H2_, which renders them unable to meet the requirements for the HER. The construction of heterostructures using Sc_2_CF_2_, Sc_2_CCl_2_, and Sc_2_CBr_2_ not only addresses the mentioned deficiency of materials but also shows significant potential for photocatalytic and optoelectronic applications. Zhang et al. investigated the electrical and optical properties of Sc_2_CF_2_/WSSe heterostructures and found that they have the potential for water splitting [33]. In addition, Sun et al. revealed that the PCE of the Sc_2_CCl_2_/SiS_2_ heterostructure can reach 23.20%, indicating promising prospects for application in the field of solar cells [34]. It is noteworthy that the VBM and CBM of the Sc_2_CF_2_ monolayer are higher than those of the Sc_2_CCl_2_ (or Sc_2_CBr_2_) monolayer, and the VBM and CBM of the Sc_2_CBr_2_ monolayer are higher than those of the Sc_2_CCl_2_ monolayer. This indicates that the Sc_2_CF_2_/Sc_2_CCl_2_, Sc_2_CF_2_/Sc_2_CBr_2_, and Sc_2_CCl_2_/Sc_2_CBr_2_ heterostructures may have a type-II band alignment. In addition, the CBM in Sc_2_CCl_2_ (or Sc_2_CBr_2_) and the VBM in Sc_2_CF_2_ are very close. This suggests that photogenerated carrier transfer in the Sc_2_CF_2_/Sc_2_CCl_2_ and Sc_2_CF_2_/Sc_2_CBr_2_ heterostructures may follow the Z-scheme pathway. Therefore, it is worthwhile to study the Sc_2_CF_2_/Sc_2_CCl_2_, Sc_2_CF_2_/Sc_2_CBr_2_, and Sc_2_CCl_2_/Sc_2_CBr_2_ heterostructures. Their potential applications in photocatalytic water splitting and solar cells show great promise.

In this paper, three types of monolayers, namely Sc_2_CF_2_, Sc_2_CCl_2_, and Sc_2_CBr_2_, were successfully vertically stacked to create Sc_2_CF_2_/Sc_2_CCl_2_, Sc_2_CF_2_/Sc_2_CBr_2_, and Sc_2_CCl_2_/Sc_2_CBr_2_ heterostructures. The stacking geometries, electronic, and optical properties of the heterostructures have been systematically studied based on first-principles calculations. According to band edge alignment and charge carrier transfer processes, the Sc_2_CF_2_/Sc_2_CCl_2_ and Sc_2_CF_2_/Sc_2_CBr_2_ heterostructures were found to have a direct Z-scheme band alignment, making them promising for photocatalytic water splitting applications. On the other hand, the Sc_2_CCl_2_/Sc_2_CBr_2_ heterostructure showed potential for use in solar cells, with a notable PCE of 20.78%. The present findings indicate that Sc_2_CX_2_/Sc_2_CY_2_ (X, Y = F, Cl, Br) heterostructures have the potential for application in solar energy conversion.

## 2. Computation Details

In this paper, all calculations are carried out using the projection enhanced wave method based on density functional theory (DFT) [35], as implemented in the Vienna Ab initio Simulation Package (VASP5.4.4) [36]. Electron–ion interactions were explained using the projected augmented wave pseudopotential (PAW), while the exchange potential and the correlation potential were described using the generalized gradient approximation (GGA) with Perdew–Burke–Ernzerhof (PBE) functional [37]. The valence electron configurations of Sc, C, F, Cl, and Br atoms are 3p^6^3d^1^4s^2^, 2s^2^2p^2^, 2s^2^2p^5^, 3s^2^3p^5^, and 4s^2^4p^5^, respectively. The energy cutoff for obtaining the relaxed lattice vector and atomic positions was set to 500 eV. All geometrical structures were relaxed until the forces and energy on each atom converged to 0.01 eV Å^−1^ and 10^−5^ eV, respectively. For the calculation of heterostructures, we utilized the DFT-D3 method to treat the interlayer vdW interaction [38]. The K-point grid for energy convergence was set to 15 × 15 × 1 for structural optimization. A vacuum layer of 20 Å was arranged along the z-axis to eliminate interactions between adjacent layers. The Heyd–Scuseria–Ernzerh (HSE06) hybrid functional was used to calculate accurate electronic and optical properties [39]. The thermal stability of the Sc_2_CF_2_/Sc_2_CCl_2_, Sc_2_CF_2_/Sc_2_CBr_2_, and Sc_2_CCl_2_/Sc_2_CBr_2_ heterostructures was further evaluated through ab initio molecular dynamics (AIMD) simulations with the NVT ensemble [40,41]. AIMD simulations were performed using a 4 × 4 × 1 supercell at 300 K. In our AIMD simulation, a total simulation time of 6 ps with a time step of 1 fs was set.

## 3. Results and Discussion

**Figure 1:**
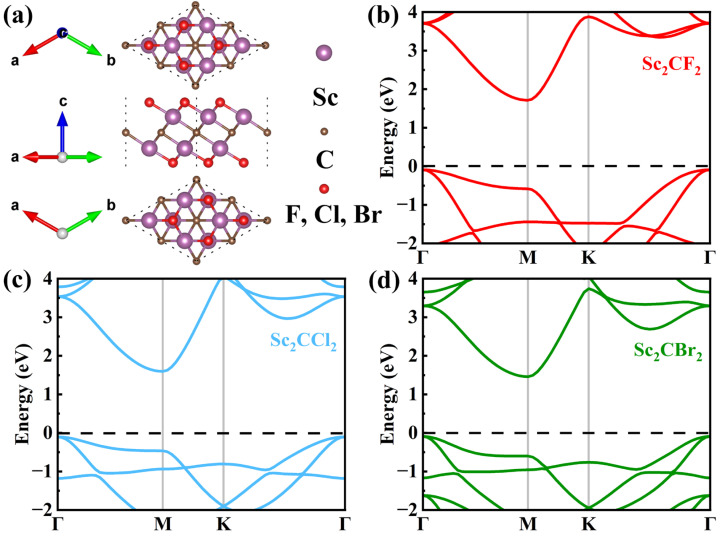
(**a**) Top view, side view, and bottom view of single-layer Sc_2_CX_2_ (X = F, Cl, Br). The band structures of (**b**) Sc_2_CF_2_, (**c**) Sc_2_CCl_2_, and (**d**) Sc_2_CBr_2_ monolayers.

The structural parameters and electronic properties of Sc_2_CX_2_ (X = F, Cl, Br) were initially studied. The atomic structures of optimized Sc_2_CX_2_ (X = F, Cl, Br) monolayers are displayed in Figure 1a. The lattice constant of the Sc_2_CF_2_ monolayer was determined to be 3.235 Å, which closely matches the theoretical value of 3.26 Å, as reported by Khang et al. [42]. The corresponding result of 3.422/3.499 Å for the Sc_2_CCl_2_/Sc_2_CBr_2_ monolayer is close to the previous theoretical value of 3.42/3.507 Å [31,43]. When the surface groups change from F to Br, the lattice parameters increase slightly due to the increase in the halogen atomic radius [31]. In addition, the band structures of the Sc_2_CX_2_ (X = F, Cl, Br) monolayers were calculated using the HSE06 method, as displayed in Figure 1b–d. It can be distinctly observed that the band shapes are fundamentally the same, despite the differences in band gap values. Moreover, we can observe that Sc_2_CX_2_ (X = F, Cl, Br) monolayers are all indirect band gap semiconductors. The CBM and VBM of the Sc_2_CX_2_ (X = F, Cl, Br) monolayers are located at the M point and Γ point, with corresponding band gaps of 1.80 eV, 1.70 eV, and 1.55 eV, respectively. All band gap values are in good agreement with the earlier reports, with percentage differences of less than 2% [32,44,45]. The results verify the rationality of our approach and parameterization.

**Figure 2:**
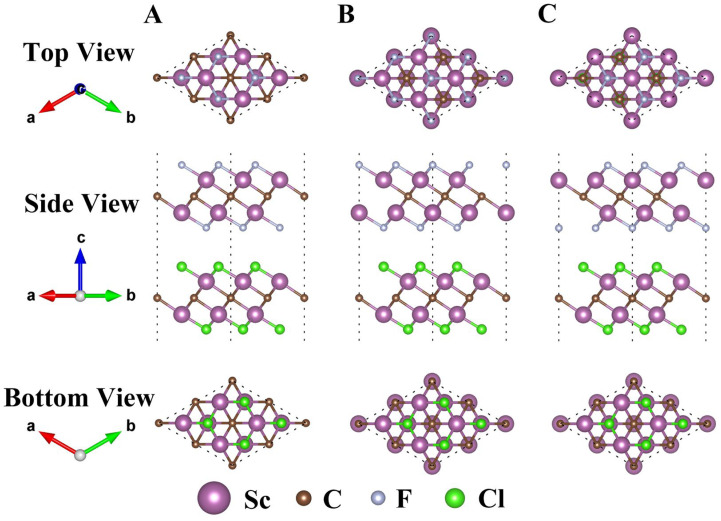
Top, side, and bottom views of the Sc_2_CF_2_/Sc_2_CCl_2_ heterostructure with three different stacking configurations of A, B, and C. The stacking configurations of Sc_2_CF_2_/Sc_2_CBr_2_ and Sc_2_CCl_2_/Sc_2_CBr_2_ heterostructures are similar to those of the Sc_2_CF_2_/Sc_2_CCl_2_ heterostructure.

Then, the structural properties of Sc_2_CF_2_/Sc_2_CCl_2_, Sc_2_CF_2_/Sc_2_CBr_2_, and Sc_2_CCl_2_/Sc_2_CBr_2_ heterostructures were researched in pursuit of the most stable configuration. There are three typical stacking configurations for all three heterostructures, i.e., A, B, and C, as illustrated in Figure 2. The structure coordinate information (POSCAR) is provided in Table S1. Table 1 presents various parameters associated with different stackings. For each heterostructure, the lattice constants of the three configurations closely match the lattice constants of the corresponding monolayer. In order to assess the stability of the heterostructures and determine the most stable configurations, the binding energy (*E*_b_) values of all configurations are computed as follows:

$$E_{b}=\frac{E_{H}-E_{{Sc}_{2}CX_{2}}-E_{{Sc}_{2}CY_{2}}}{S_{0}}$$

where $E_{H}$ represents the energy of the Sc_2_CF_2_/Sc_2_CCl_2_, Sc_2_CF_2_/Sc_2_CBr_2_, and Sc_2_CCl_2_/Sc_2_CBr_2_ heterostructures, respectively. Here, $S_{0}$ represents the interface area, while $E_{{Sc}_{2}CX_{2}}$ and $E_{{Sc}_{2}CY_{2}}$ represent the energy of the Sc_2_CF_2_, Sc_2_CCl_2_, and Sc_2_CBr_2_ monolayers, respectively. From Table 1, we can see that the minus *E*_b_ values for all stacking configurations manifest that the interface formation is exothermic, which is favorable for their preparation [46]. Clearly, for Sc_2_CF_2_/Sc_2_CCl_2_, Sc_2_CF_2_/Sc_2_CBr_2_, and Sc_2_CCl_2_/Sc_2_CBr_2_ heterostructures, stacking-B exhibits the smallest *E*_b_ of −35.67 meV∙Å^−2^, −28.53 meV∙Å^−2^, and −19.96 meV∙Å^−2^, indicating that stacking-B is the most stable among the three stacking configurations. In addition, this value is smaller than the previously reported C_2_N/ZnSe heterostructure (−12.1 meV∙Å^−2^) [47] and BiTeCl/GeSe heterostructure (−11.07 meV∙Å^−2^) [48], revealing that Sc_2_CF_2_/Sc_2_CCl_2_, Sc_2_CF_2_/Sc_2_CBr_2_, and Sc_2_CCl_2_/Sc_2_CBr_2_ are vdW heterostructures. Thus, only the stacking-B heterostructure was taken into consideration in all the following calculations. Indispensably, AIMD simulations are performed to validate the thermodynamic stability of the heterostructure. As depicted in Figure S1, the geometrical structures of the Sc_2_CF_2_/Sc_2_CCl_2_, Sc_2_CF_2_/Sc_2_CBr_2_, and Sc_2_CCl_2_/Sc_2_CBr_2_ heterostructures remained stable during the 6 ps simulation at a temperature of 300 K. No bonds were broken, and the energy fluctuation was minimal, indicating that each heterostructure is sufficiently stable at room temperature. Furthermore, to verify the dynamical stability of the Sc_2_CF_2_/Sc_2_CCl_2_, Sc_2_CF_2_/Sc_2_CBr_2_, and Sc_2_CCl_2_/Sc_2_CBr_2_ heterostructures, we calculated their phonon spectrum with a 3 × 3 × 1 supercell and implemented them in the PHONOPY code with the density functional perturbation theory (DFPT), as shown in Figure S2. It can be seen that there are some insignificant imaginary frequencies near the G-point. This phenomenon also exists in the phonon spectra of some experimentally prepared 2D materials, but the imaginary frequency near the G-point can be ignored [49,50,51]. This phenomenon may be attributed to inadequate computational accuracy, which can be eliminated by creating a larger supercell or setting a higher parameter accuracy. Thus, the Sc_2_CF_2_/Sc_2_CCl_2_, Sc_2_CF_2_/Sc_2_CBr_2_, and Sc_2_CCl_2_/Sc_2_CBr_2_ heterostructures are dynamically stable.

**Figure 3:**
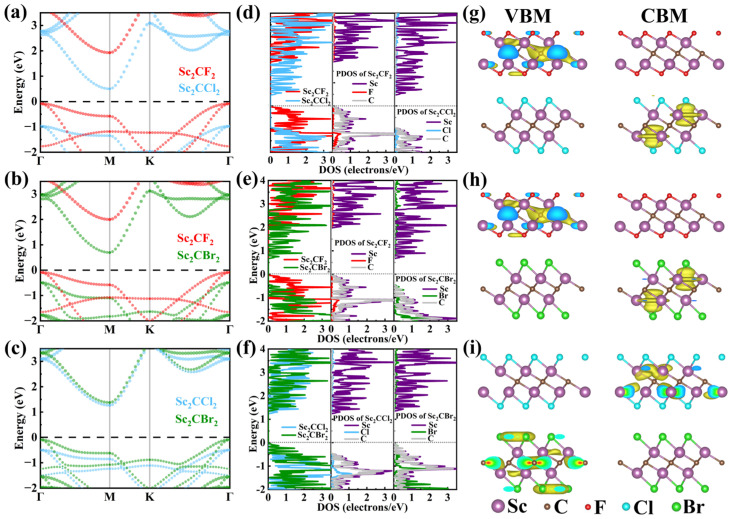
The projected band alignments of the (**a**) Sc_2_CF_2_/Sc_2_CCl_2_ heterostructure, (**b**) Sc_2_CF_2_/Sc_2_CBr_2_ heterostructure, and (**c**) Sc_2_CCl_2_/Sc_2_CBr_2_ heterostructures. (**d**–**f**) The PDOS of the Sc_2_CF_2_/Sc_2_CCl_2_, Sc_2_CF_2_/Sc_2_CBr_2_, and Sc_2_CCl_2_/Sc_2_CBr_2_ heterostructures. (**g**–**i**) The visualization of band decomposed charge density for Sc_2_CF_2_/Sc_2_CCl_2_, Sc_2_CF_2_/Sc_2_CBr_2_, and Sc_2_CCl_2_/Sc_2_CBr_2_ heterostructures, respectively.

The projected band structures of Sc_2_CF_2_/Sc_2_CCl_2_, Sc_2_CF_2_/Sc_2_CBr_2_, and Sc_2_CCl_2_/Sc_2_CBr_2_ heterostructures were calculated based on the HSE06 hybrid functional, as depicted in Figure 3a–c. It can be found that the Sc_2_CF_2_/Sc_2_CCl_2_, Sc_2_CF_2_/Sc_2_CBr_2_, and Sc_2_CCl_2_/Sc_2_CBr_2_ heterostructures all show the characteristics of semiconductors with indirect band structures. The VBM and CBM are located at the M point and Γ point, with band gaps of 0.58 eV, 0.78 eV, and 1.35 eV, respectively. Compared with the band gaps of monolayers, the significantly reduced band gaps of heterostructures are due to the interaction of vdW forces, which lead to a change in the band structure upon contact [21]. It should be noted that the smaller band gap of Sc_2_CF_2_/Sc_2_CCl_2_ and Sc_2_CF_2_/Sc_2_CBr_2_ heterostructures can lead to improved optical absorption performance during the photocatalytic reaction process. In addition, we can clearly see that the VBM and CBM of the three heterostructures are each occupied by two monolayers, demonstrating an inherent type-II heterostructure. Among them, the VBM of Sc_2_CF_2_/Sc_2_CCl_2_ and Sc_2_CF_2_/Sc_2_CBr_2_ heterostructures is mainly attributed to the Sc_2_CF_2_ monolayer, while the CBM mainly comes from the Sc_2_CCl_2_ (or Sc_2_CBr_2_) monolayer. Hence, electrons mainly occupy Sc_2_CCl_2_ (or Sc_2_CBr_2_), while holes mainly occupy Sc_2_CF_2_. Similarly, the VBM of the Sc_2_CCl_2_/Sc_2_CBr_2_ heterostructure is mainly contributed by the Sc_2_CBr_2_ layer, whereas the CBM is entirely dominated by the Sc_2_CCl_2_ layer. It is certain that the type-II band structures can separate the photoexcited electrons and holes into different monolayers, which is conducive to reducing the carrier recombination rate. This separation can improve the utilization of photogenerated carriers and extend their lifetime [44].

In addition, Figure 3d–f shows the projected density of states (PDOS) of the Sc_2_CF_2_/Sc_2_CCl_2_, Sc_2_CF_2_/Sc_2_CBr_2_, and Sc_2_CCl_2_/Sc_2_CBr_2_ heterostructures, respectively. From Figure 3d, it can be seen that in the Sc_2_CF_2_/Sc_2_CCl_2_ heterostructure, the peak with the highest energy below the Fermi level mainly originates from the Sc and C atoms in Sc_2_CF_2_, while the peak with the lowest energy above the Fermi level is mainly contributed by the Sc atom in Sc_2_CCl_2_. This shows that the VBM of the Sc_2_CF_2_/Sc_2_CCl_2_ heterostructure is contributed by the Sc_2_CF_2_, while the CBM is contributed by the Sc_2_CCl_2_. As shown in Figure 3e, the VBM of the Sc_2_CF_2_/Sc_2_CBr_2_ heterostructure is mainly contributed by the Sc and C atoms of Sc_2_CF_2_, while the CBM mainly comes from the Sc atom of Sc_2_CBr_2_. This indicates that the VBM of the Sc_2_CF_2_/Sc_2_CBr_2_ heterostructure comes from the electronic states of Sc_2_CF_2_, while the CBM comes from the electronic states of Sc_2_CBr_2_. In Figure 3f, we can clearly observe that the CBM of the Sc_2_CCl_2_/Sc_2_CBr_2_ configuration is contributed by the Sc atom of Sc_2_CCl_2_. However, the VBM is not only contributed by the Sc and C atoms but also by the Br atom. This shows that the VBM of the Sc_2_CCl_2_/Sc_2_CBr_2_ heterostructure originates from the Sc_2_CBr_2_ monolayer, while the CBM comes from the Sc_2_CCl_2_ monolayer. In addition, the orbitals of the C atom and Sc atom are completely hybridized. This PDOS result further confirms that the CBM and VBM of Sc_2_CF_2_/Sc_2_CCl_2_, Sc_2_CF_2_/Sc_2_CBr_2_, and Sc_2_CCl_2_/Sc_2_CBr_2_ heterostructures are located on different monolayers.

In Figure 3g–i, we displayed the band decomposed charge densities of the VBM and CBM in Sc_2_CF_2_/Sc_2_CCl_2_, Sc_2_CF_2_/Sc_2_CBr_2_, and Sc_2_CCl_2_/Sc_2_CBr_2_ heterostructures, respectively. In Sc_2_CF_2_/Sc_2_CCl_2_ and Sc_2_CF_2_/Sc_2_CBr_2_ heterostructures, it can be observed that the VBM is located in Sc_2_CF_2_, while the CBM is located in Sc_2_CCl_2_ (or Sc_2_CBr_2_). Consistent with the above analysis, the VBM and CBM of the Sc_2_CCl_2_/Sc_2_CBr_2_ heterostructure are located on the lower layer (Sc_2_CBr_2_) and upper layer (Sc_2_CCl_2_), respectively. There is no charge density overlap between the VBM and CBM, indicating that heterostructures like Sc_2_CF_2_/Sc_2_CCl_2_, Sc_2_CF_2_/Sc_2_CBr_2_, and Sc_2_CCl_2_/Sc_2_CBr_2_ can effectively separate electrons and holes [52].

The above analysis shows that the Sc_2_CF_2_/Sc_2_CCl_2_, Sc_2_CF_2_/Sc_2_CBr_2_, and Sc_2_CCl_2_/Sc_2_CBr_2_ heterostructures exhibit staggered type-II band alignment. This structure can promote the effective separation of holes and electrons, reduce the carrier recombination rate, and play an important role in photocatalytic water splitting and optoelectronic devices.

**Figure 4:**
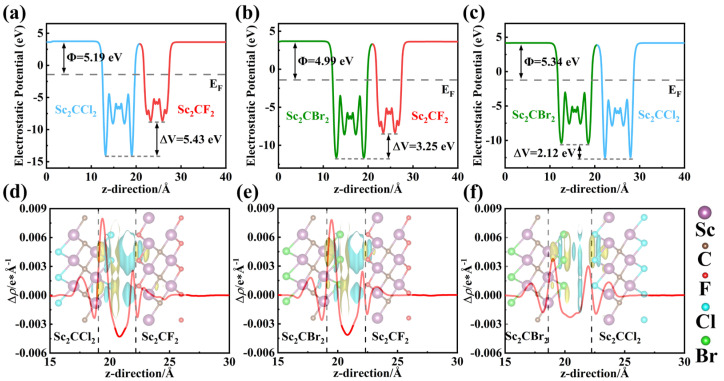
(**a**–**c**) The electrostatic potential along the z-axis direction of Sc_2_CF_2_/Sc_2_CCl_2_, Sc_2_CF_2_/Sc_2_CBr_2_, and Sc_2_CCl_2_/Sc_2_CBr_2_ heterostructures. (**d**–**f**) The plane-averaged charge density difference of Sc_2_CF_2_/Sc_2_CCl_2_, Sc_2_CF_2_/Sc_2_CBr_2_, and Sc_2_CCl_2_/Sc_2_CBr_2_ heterostructures. The insert is the 3D view of the charge density difference, where the yellow and blue represent the regions of electron accumulation and depletion, respectively.

The difference in work functions between two semiconductors can lead to charge redistribution and the formation of an electric field at the interface. This electric field will determine the transfer process of photogenerated charges. Thus, the work functions of the Sc_2_CF_2_, Sc_2_CCl_2_, and Sc_2_CBr_2_ monolayers, as well as the Sc_2_CF_2_/Sc_2_CCl_2_, Sc_2_CF_2_/Sc_2_CBr_2_, and Sc_2_CCl_2_/Sc_2_CBr_2_ heterostructures, are calculated using the following formula:

$$\Phi =E_{vac}-E_{F}$$

in which $E_{vac}$ and $E_{F}$ represent the vacuum level and Fermi level, respectively. As shown in Figure S3a–c, Sc_2_CF_2_, Sc_2_CCl_2_, and Sc_2_CBr_2_ monolayers exhibit a fixed work function of 5.02 eV, 5.86 eV, and 5.48 eV, respectively, due to their highly symmetrical crystal structure [28]. Compared to Sc_2_CCl_2_ and Sc_2_CBr_2_ monolayers, the Sc_2_CF_2_ monolayer exhibits a smaller work function and a higher Fermi level. Thus, in the Sc_2_CF_2_/Sc_2_CCl_2_ and Sc_2_CF_2_/Sc_2_CBr_2_ heterostructures, free electrons can migrate from Sc_2_CF_2_ to Sc_2_CCl_2_ (or Sc_2_CBr_2_) until their Fermi levels reach equilibrium. As shown in Figure 4a,b, the work functions of the Sc_2_CF_2_/Sc_2_CCl_2_ and Sc_2_CF_2_/Sc_2_CBr_2_ heterostructures are 5.19 eV and 4.99 eV, respectively. At the same time, there are potential drops of 5.43 eV and 3.25 eV at the Sc_2_CF_2_/Sc_2_CCl_2_ and Sc_2_CF_2_/Sc_2_CBr_2_ heterostructures, indicating the presence of a built-in electric field at the interface of the heterostructures [52]. It also indicates that electrons are inclined to flow to Sc_2_CCl_2_ (or Sc_2_CBr_2_) monolayers. The built-in electric field will create a driving force to promote the combination of photogenerated electron–hole pairs between the electrons in the CBM of Sc_2_CCl_2_ (or Sc_2_CBr_2_) and the holes in the VBM of Sc_2_CF_2_. As displayed in Figure 4c, the difference in monolayer work function leads to the transfer of electrons from Sc_2_CBr_2_ to Sc_2_CCl_2_, causing a decline in the Fermi level in Sc_2_CCl_2_ and Sc_2_CBr_2_. The work function of the heterostructure in the final equilibrium state is 5.34 eV. Moreover, a potential drop of 2.12 eV is found across the interface. This is proof of a built-in electric field at the interface of the Sc_2_CCl_2_/Sc_2_CBr_2_ heterostructure.

During the formation of a heterostructure, the charge near the interface will be redistributed due to the presence of interlayer interactions. In order to explore the charge transfer mechanism of Sc_2_CF_2_/Sc_2_CCl_2_, Sc_2_CF_2_/Sc_2_CBr_2_, and Sc_2_CCl_2_/Sc_2_CBr_2_ heterostructures, the planar averaged charge density difference and 3D differential charge density difference were calculated using the following equation:

$$∆\rho =\rho_{het}-\rho_{SCX}-\rho_{SCY},$$

where the $\rho_{het}$ stand for the density of Sc_2_CF_2_/Sc_2_CCl_2_, Sc_2_CF_2_/Sc_2_CBr_2_, and Sc_2_CCl_2_/Sc_2_CBr_2_ heterostructures, and the $\rho_{SCX}$ and $\rho_{SCY}$ represent the corresponding densities of Sc_2_CF_2_, Sc_2_CCl_2_, and Sc_2_CBr_2_ monolayers. As shown in Figure 4d,e, for Sc_2_CF_2_/Sc_2_CCl_2_ and Sc_2_CF_2_/Sc_2_CBr_2_, it can be clearly seen that a large number of negative charges are assembled in the side of Sc_2_CCl_2_ (or Sc_2_CBr_2_) monolayers, while positive charges cluster on the side of Sc_2_CF_2_. This leads to the formation of a built-in electric field from Sc_2_CF_2_ to Sc_2_CCl_2_ (or Sc_2_CBr_2_). As shown in Figure 4f, the electrons at the interface are depleted near the Sc_2_CBr_2_ monolayer and accumulate at the Sc_2_CCl_2_ monolayers, forming a built-in electric field from Sc_2_CBr_2_ to Sc_2_CCl_2_. In addition, the Bader charges obtained indicate that about 0.0072 |e| (0.0052 |e|) are transferred from the Sc_2_CF_2_ monolayer to the Sc_2_CCl_2_ (or Sc_2_CBr_2_) monolayers in the case of the Sc_2_CF_2_/Sc_2_CCl_2_ (Sc_2_CF_2_/Sc_2_CBr_2_) heterostructure. Furthermore, around 0.0018 |e| is transferred from Sc_2_CBr_2_ to Sc_2_CCl_2_ within the Sc_2_CCl_2_/Sc_2_CBr_2_ heterostructure.

**Figure 5:**
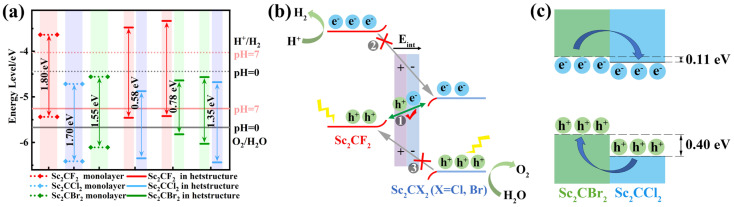
(**a**) The band position of monolayers and heterostructures. (**b**) Charge transfer mechanism of Sc_2_CF_2_/Sc_2_CX_2_ (X = Cl, Br). (**c**) Schematic diagram illustrating the migration of photogenerated electrons and holes at the Sc_2_CCl_2_/Sc_2_CBr_2_ heterostructure.

In addition to the band gap value, the band edge alignment is also a crucial parameter for evaluating the application of the heterostructure. Therefore, we computed the band alignments of Sc_2_CF_2_, Sc_2_CCl_2_, and Sc_2_CBr_2_ monolayers, as well as the Sc_2_CF_2_/Sc_2_CCl_2_, Sc_2_CF_2_/Sc_2_CBr_2_, and Sc_2_CCl_2_/Sc_2_CBr_2_ heterostructures, using the method suggested by Toroker et al. [53]. Figure 5a reveals that the VBM of the Sc_2_CF_2_ monolayer exceeds that of E_O2/H2O_. The Sc_2_CCl_2_ and Sc_2_CBr_2_ monolayers exhibit very similar characteristics in their band edge alignments, with both CBM being lower than the energy level of E_H+/H2_. Based on the aforementioned analysis, the band positions of the Sc_2_CF_2_, Sc_2_CCl_2_, and Sc_2_CBr_2_ monolayers are unsuitable for photocatalysis. For the Sc_2_CF_2_/Sc_2_CCl_2_ and Sc_2_CF_2_/Sc_2_CBr_2_ heterostructures, the VBM and CBM of the Sc_2_CF_2_ layer are higher than those of the Sc_2_CCl_2_ (or Sc_2_CBr_2_) layer, further affirming that the heterostructure exhibits a type-II band alignment. For the Sc_2_CCl_2_/Sc_2_CBr_2_ heterostructure, both the VBM and CBM of Sc_2_CBr_2_ exceed those of the Sc_2_CCl_2_ layer, indicating a type-II band alignment. The CBM of Sc_2_CCl_2_ is lower than that of E_H+/H2_, making the Sc_2_CCl_2_/Sc_2_CBr_2_ heterostructure unsuitable for photocatalytic water splitting reactions.

The photocatalytic water splitting reaction mechanism of Sc_2_CF_2_/Sc_2_CX_2_ (X = Cl, Br) is shown in Figure 5b. In general, three possible processes are considered here: ① The photoexcited holes at the VBM of Sc_2_CF_2_ recombine with electrons at the CBM of Sc_2_CCl_2_ (or Sc_2_CBr_2_), which represents a direct Z-scheme transfer path (indicated by the green line with double-headed arrows). ②–③ Photogenerated electrons at the CBM of Sc_2_CF_2_ migrate to the CBM of Sc_2_CCl_2_ (or Sc_2_CBr_2_), while photogenerated holes at the VBM of Sc_2_CCl_2_ (or Sc_2_CBr_2_) migrate to the VBM of Sc_2_CF_2_. This migration follows a traditional type-II path (indicated by gray lines with arrows). Electronic property analysis shows that the band alignments of the Sc_2_CF_2_/Sc_2_CX_2_ (X = Cl, Br) heterostructure are made up of the CBM of the Sc_2_CCl_2_ (or Sc_2_CBr_2_) layer and the VBM of the Sc_2_CF_2_ layer. Compared to the band gap of two monolayers, the heterostructure has a smaller band gap (Figure 5a), indicating a higher rate of photogenerated electron–hole pair recombination at the interface compared to the rate of intralayer recombination. Meanwhile, due to the built-in electric field from Sc_2_CF_2_ to Sc_2_CCl_2_ (or Sc_2_CBr_2_), the recombination of photogenerated electrons in the CBM of Sc_2_CCl_2_ (or Sc_2_CBr_2_) and photogenerated holes in the VBM of Sc_2_CF_2_ is accelerated, promoting the recombination of path ① carriers. In addition, electrons have varying additional potential energies at different points in the space charge region, a phenomenon known as energy band bending [54]. The positive charge on the Sc_2_CCl_2_ (or Sc_2_CBr_2_) is repelled by the holes on the Sc_2_CF_2_, causing the energy band to bend downward. Correspondingly, as the electrons move, the energy bands of the Sc_2_CF_2_ bend upward, forming a potential barrier at the interface. Due to the presence of built-in electric fields and potential barriers, the transfer of electrons from the CBM of Sc_2_CF_2_ to the CBM of Sc_2_CCl_2_ (or Sc_2_CBr_2_), as well as the transfer of holes from the VBM of Sc_2_CCl_2_ (or Sc_2_CBr_2_) to the VBM of Sc_2_CF_2_, are suppressed. Therefore, electron transfer in paths ② and ③ is repressed. After absorbing photon energy, the electrons are excited to the CBM, while the holes remain in the VBM. Due to the obstruction of path ② and path ③, photogenerated electrons gather in the CBM of Sc_2_CF_2_, while photogenerated holes gather in the VBM of Sc_2_CCl_2_ (or Sc_2_CBr_2_), which facilitates the efficient separation of photogenerated carriers and prolongs their lifetime [23]. Therefore, it is difficult for electrons and holes to transfer following the type-II pathway, and the Sc_2_CF_2_/Sc_2_CX_2_ heterostructure should be used as the photocatalyst for the Z-scheme. According to the above analysis, the Sc_2_CF_2_ layer exhibits a higher reduction ability. Photogenerated electrons and hydrogen ions undergo a reduction reaction on the CBM of the Sc_2_CF_2_ layer to produce hydrogen. Meanwhile, in the highly oxidizing Sc_2_CCl_2_ (or Sc_2_CBr_2_) layer, the photogenerated holes on the VBM react with hydroxyl groups to produce oxygen, thereby improving the photocatalytic performance.

Differently, the Sc_2_CCl_2_/Sc_2_CBr_2_ heterostructure is not suitable as a photocatalyst due to the fact that the CBM is lower than the energy level of E_H+/H2_ (Figure 5a). However, they can function as absorption layers for solar cells. As shown in Figure 5c, the conduction band offset and valence band offset between the Sc_2_CCl_2_ and Sc_2_CBr_2_ layers are 0.11 eV and 0.40 eV, respectively. Therefore, under the influence of valence band offset, the photogenerated holes in the Sc_2_CCl_2_ layer tend to jump to the VBM of the Sc_2_CBr_2_ layer. Simultaneously, due to the lower CBM energy of Sc_2_CCl_2_ in the Sc_2_CCl_2_/Sc_2_CBr_2_ heterostructure, photogenerated electrons tend to move to the CBM of Sc_2_CCl_2_, resulting in a type-II band alignment. The very small conduction band offset can improve the energy conversion efficiency of the solar cell, while the large valence band offset limits the electrons in the Sc_2_CCl_2_ monolayer and the holes in the Sc_2_CBr_2_ monolayer [55]. Therefore, the rate of electron hole recombination will decrease, and the lifetime of photogenerated carriers will be extended. This will promote the formation of indirect excitons, which can be utilized in optoelectronic devices.

**Figure 6:**
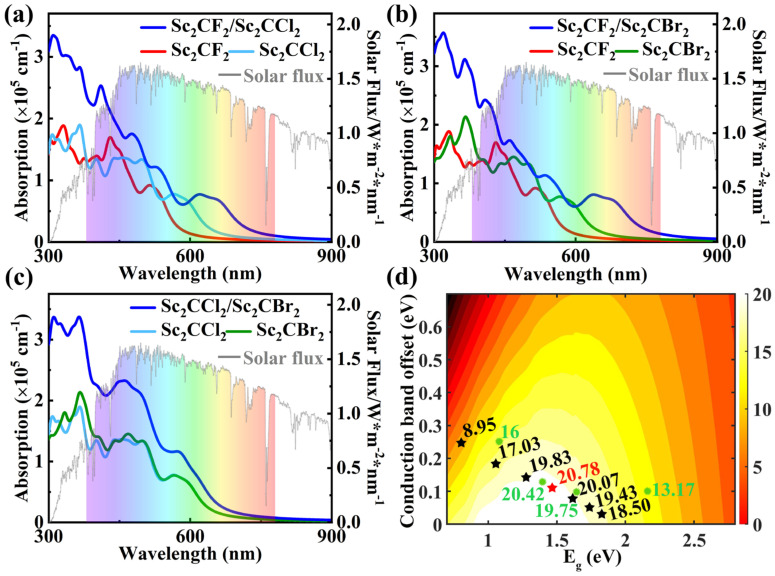
(**a**–**c**) Optical absorption coefficient as a function of energy for the Sc_2_CF_2_/Sc_2_CCl_2_, Sc_2_CF_2_/Sc_2_CBr_2_, and Sc_2_CCl_2_/Sc_2_CBr_2_ heterostructures, along with their respective isolated monolayers. (**d**) PCE as a function of donor band gap and conduction band offset of the Sc_2_CCl_2_/Sc_2_CBr_2_ heterostructure.

Considering that the construction of vdW heterostructures is an effective approach to enhance optical absorption and achieve excellent photovoltaic performance, therefore, to analyze the optical properties of the Sc_2_CF_2_/Sc_2_CCl_2_, Sc_2_CF_2_/Sc_2_CBr_2_, and Sc_2_CCl_2_/Sc_2_CBr_2_ heterostructures, we calculated the optical absorption of the Sc_2_CF_2_ monolayer, Sc_2_CCl_2_ monolayer, Sc_2_CBr_2_ monolayer, and the Sc_2_CF_2_/Sc_2_CCl_2_, Sc_2_CF_2_/Sc_2_CBr_2_, and Sc_2_CCl_2_/Sc_2_CBr_2_ heterostructures, as shown in Figure 6a–c. Among them, the optical absorption coefficient is determined by the following equation [56]:

$$\alpha \left(\omega \right)=\sqrt{2}\omega \sqrt{\sqrt{\varepsilon_{1}^{2}\left(\omega \right)+\varepsilon_{2}^{2}\left(\omega \right)}-\varepsilon_{1}(\omega )}$$

where $\varepsilon_{1}(\omega )$ and $\varepsilon_{2}(\omega )$ represent the real and imaginary parts of the complex dielectric function ε(ω), respectively. As illustrated in Figure 6a, we found that, compared to monolayers Sc_2_CF_2_ and Sc_2_CCl_2_, the Sc_2_CF_2_/Sc_2_CCl_2_ heterostructure has a wide absorption range from UV light to visible light due to its reduced band gap. It can be seen that the optical absorption coefficient of the Sc_2_CF_2_/Sc_2_CCl_2_ heterostructure is much larger than that of Sc_2_CF_2_ and Sc_2_CCl_2_ in both the UV and visible light ranges. More importantly, the Sc_2_CF_2_/Sc_2_CCl_2_ heterostructure shows a high absorption coefficient in the visible light region, reaching up to 2.53 × 10^5^ cm^−1^ at a wavelength of 410 nm. The enhancement of the optical absorption coefficient is mainly due to the interlayer coupling between two monolayers of the Sc_2_CF_2_/Sc_2_CCl_2_ heterostructure [57]. Therefore, it is expected that the Sc_2_CF_2_/Sc_2_CCl_2_ heterostructure can act as an efficient visible light-harvesting photocatalyst. As can be seen from Figure 6b, compared with the Sc_2_CF_2_ monolayer and Sc_2_CBr_2_ monolayer, the significantly increased optical absorption in the UV and visible light regions of the Sc_2_CF_2_/Sc_2_CBr_2_ heterostructure is due to the interlayer coupling [58]. At the same time, compared with the Sc_2_CF_2_ monolayer and Sc_2_CBr_2_ monolayer, the increase in the optical absorption range of the Sc_2_CF_2_/Sc_2_CBr_2_ heterostructure is on account of the decrease in the band gap. Therefore, compared to the Sc_2_CF_2_ and Sc_2_CBr_2_ monolayers, the Sc_2_CF_2_/Sc_2_CBr_2_ heterostructure exhibits superior optical absorption performance, enabling efficient solar energy harvesting.

Light-absorbing materials not only need to have a suitable electronic structure but also need to have the ability to harvest solar light. Therefore, it is of great significance to study the optical properties of the Sc_2_CCl_2_/Sc_2_CBr_2_ heterostructure. The calculated absorption spectra of Sc_2_CCl_2_ and Sc_2_CBr_2_ monolayers, as well as the Sc_2_CCl_2_/Sc_2_CBr_2_ heterostructure, are shown in Figure 6c. In the UV and visible regions, the absorption intensity of Sc_2_CCl_2_ and Sc_2_CBr_2_ monolayers is weak. However, the absorption peak of the Sc_2_CCl_2_/Sc_2_CBr_2_ heterostructure in the visible region is nearly 2.33 × 10^5^ cm^−1^, which is 1.71 times that of the Sc_2_CCl_2_ monolayer. The enhancement of the optical absorption coefficient is mainly due to the interlayer coupling between two monolayers of the Sc_2_CCl_2_/Sc_2_CBr_2_ heterostructure [58]. Compared to both monolayers, the absorption range of the Sc_2_CCl_2_/Sc_2_CBr_2_ heterostructure increases due to its reduced band gap. Therefore, it can be concluded that the Sc_2_CCl_2_/Sc_2_CBr_2_ heterostructure would be a promising material for solar cells.

For device applications, in addition to the electronic and optical properties of the Sc_2_CCl_2_/Sc_2_CBr_2_ heterostructure analyzed above, such as limited band gaps, strong solar light-harvesting capabilities, and easy separation of electrons and holes with type-II band alignment, the ability to convert photon energy into electricity is also critical for solar cell applications. We use the method developed by Scharber et al. to calculate the PCE of solar cells, and its formula is as follows [59]:

$$\eta =\frac{J_{sc}V_{oc}\beta_{FF}}{P_{solar}}=\frac{0.65(E_{g}^{d}-\Delta E_{c}-0.3)\int_{E_{g}^{d}}^{\infty }\frac{J_{Ph\left(\hbar \omega \right)}}{\hbar \omega }d(\hbar \omega )}{\int_{0}^{\infty }J_{Ph}\left(\hbar \omega \right)d(\hbar \omega )}$$

where 0.65 represents the band fill factor, $E_{g}^{d}$ stands for the optical band gap of the donor, and $\Delta E_{c}$ represents the conduction band offset (CBO). The open circuit voltage is $E_{g}^{d}-\Delta E_{c}-0.3$, and $J_{Ph}\left(\hbar \omega \right)$ is the 1.5 AM solar energy flux at the photon energy $\left(\hbar \omega \right)$. As shown in Figure 6d, the calculated PCE of the Sc_2_CCl_2_/Sc_2_CBr_2_ heterostructure is about 20.78% (highlighted by the red star), which surpasses that of many other heterostructures, such as GeSe/AsP (16%) [13], InS/InSe (13.17%) [60], Hf_2_CO_2_/MoS_2_ (19.75%) [29], and MoS_2_/BP (20.42%) [61] heterostructures (highlighted by the green circle). Thus, we conclude that the Sc_2_CCl_2_/Sc_2_CBr_2_ heterostructure is more promising and competitive for 2D vdW heterostructure solar cells.

Strain engineering is an effective method to change the structural, electronic, and magnetic properties of 2D materials [62]. In addition, strain is unavoidable in industrial production, which comes from bending, external loads, and lattice mismatch [46]. Applying a biaxial strain will alter the band structure of the heterostructure and affect its photocatalytic and photovoltaic performance [42,45]. Then, the effects of in-plane biaxial strain on the electronic properties of Sc_2_CF_2_/Sc_2_CCl_2_, Sc_2_CF_2_/Sc_2_CBr_2_, and Sc_2_CCl_2_/Sc_2_CBr_2_ heterostructures are systematically studied. Here, the inner-layer biaxial strain (ε_in_) is defined by ε_in_ = [(*L* − *L*_0_)/*L*_0_] × 100%, where *L* and *L*_0_ are the lattice constants before and after the strain application, respectively. The applied strains η are −8%, −6%, −4%, −2%, 2%, 4%, 6%, and 8%, respectively. A negative value of η means that compressive strain is applied to the heterostructure. When η is positive, it indicates that tensile strain is applied to the heterostructure.

**Figure 7:**
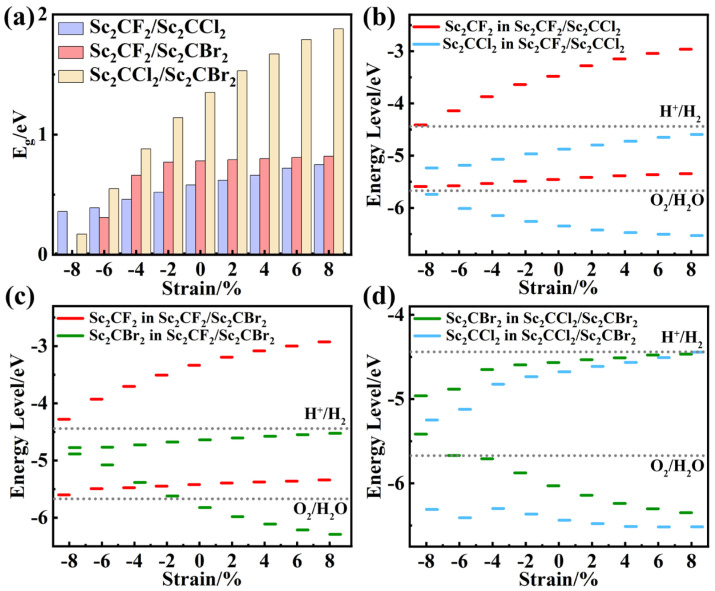
The (**a**) band gaps and (**b**–**d**) band alignment of strained Sc_2_CF_2_/Sc_2_CCl_2_, Sc_2_CF_2_/Sc_2_CBr_2_, and Sc_2_CCl_2_/Sc_2_CBr_2_ heterostructures.

As shown in Figure S4, the electronic properties of the Sc_2_CF_2_/Sc_2_CCl_2_ heterostructure are significantly changed by applying biaxial strain. Compared with the Sc_2_CF_2_/Sc_2_CCl_2_ heterostructure without strain (Figure 3a), the applied strain changes the band gap of the heterostructure. From Figure 7a, it can be seen that when the compressive strain is −8%, −6%, −4%, and −2%, the band gap of the heterostructure decreases to 0.36, 0.39 eV, 0.46 eV, and 0.52 eV, respectively. Among them, the positions of the CBM and VBM have not changed and are still located at the high symmetry points M and Γ, as shown in Figure S4a–d. When the tensile strains are +2%, +4%, +6%, and +8%, respectively, the band gaps of the heterostructure increase to 0.62 eV, 0.66 eV, 0.72 eV, and 0.75 eV, respectively. The positions of the CBM and VBM are still located at the high symmetry points M and Γ, respectively (Figure S4e–h). With the increase in strain, the CBM of the Sc_2_CCl_2_ monolayer gradually moves away from the Fermi level, causing an increase in the band gaps. It can be seen from Figure 7b that the Sc_2_CF_2_/Sc_2_CCl_2_ heterostructure maintains a type-II band alignment throughout the strain. As for the band edge, all the heterostructures maintained photocatalytic activity under strain.

The electronic properties of the Sc_2_CF_2_/Sc_2_CBr_2_ heterostructure changed significantly when biaxial strain was applied, as shown in Figure S5. In contrast to the strain-free Sc_2_CF_2_/Sc_2_CBr_2_ heterostructure (Figure 3b), applying strain not only alters the band gaps of the heterostructure but also changes the band alignment of the heterostructure. As can be seen from Figure 7a, the band gaps of the Sc_2_CF_2_/Sc_2_CBr_2_ heterostructure decrease to 0.31 eV, 0.66 eV, and 0.77 eV when the compression strain is −6%, −4%, and −2%, with the CBM and VBM located at highly symmetric points M and Γ (Figure S5b–d). However, when the compressive strain increases to −8%, the band gap of the Sc_2_CF_2_/Sc_2_CBr_2_ heterostructure decreases to 0 eV. This indicates that the heterostructure transitions from an indirect band gap semiconductor to a metal under −8% compressive strain because the CBM (VBM) moves below (above) the Fermi level, as shown in Figure S5a. When the tensile strain was +2%, +4%, +6%, and +8%, the CBM and VBM were located at highly symmetric points M and Γ, with band gaps increasing to 0.79 eV, 0.80 eV, 0.81 eV, and 0.82 eV, respectively, as shown in Figure S5e–h. From Figure 7c, when the compressive strain is between −6% and −4%, the VBM of the Sc_2_CBr_2_ layer is positioned at a higher energy level than that of the VBM of the Sc_2_CF_2_ layer. Consequently, the VBM of the Sc_2_CF_2_/Sc_2_CBr_2_ heterostructure shifts from the Sc_2_CF_2_ layer to the Sc_2_CBr_2_ layer, leading to a transition from type-II to type-I. In addition, when the compression strain is −2%, the VBM of Sc_2_CBr_2_ is higher than that of E_O2/H2O_, which is unfavorable for the photocatalytic reaction. By analyzing the band structure of the Sc_2_CF_2_/Sc_2_CBr_2_ heterostructure under strain, it is considered that the strain affects the relative position of atoms as well as the bonding properties and strength of the atoms, leading to a change in the band structure. The band alignment of the Sc_2_CF_2_/Sc_2_CBr_2_ heterostructure can be changed from type-I to type-II under different strain conditions.

For the Sc_2_CCl_2_/Sc_2_CBr_2_ heterostructure, the applied biaxial strain range is still −8%~8%. As shown in Figure S6, it is noteworthy that under −8%~6% biaxial strains, the heterostructures consistently maintain type-II banding and retain indirect band gap characteristics. As displayed in Figure 7a, the band gaps of the Sc_2_CCl_2_/Sc_2_CBr_2_ heterostructure decrease to 0.17 eV, 0.55 eV, 0.88 eV, and 1.14 eV when compressive strain is applied. As the tensile strain increases, the band gap also increases, reaching 1.53 eV, 1.67 eV, 1.79 eV, and 1.88 eV, respectively. Under −8%~6% biaxial strains, the CBM and VBM are still contributed by Sc_2_CCl_2_ and Sc_2_CBr_2_, located at the M and Γ points, respectively, as depicted in Figure 7d. Unlike these changes, under the tensile strain of 8%, the CBM of the Sc_2_CCl_2_ layer becomes higher than the CBM of the Sc_2_CBr_2_ layer. Thus, the CBM of the Sc_2_CCl_2_/Sc_2_CBr_2_ heterostructure shifts from the Sc_2_CCl_2_ layer to the Sc_2_CBr_2_ layer, leading to a type-II to type-I transformation. In addition, we calculated the PCE values of the Sc_2_CCl_2_/Sc_2_CBr_2_ heterostructure under various biaxial strains, as illustrated in Figure 6d (highlighted by the black star). From Figure S7, we can see that a maximum PCE of 20.07% can be achieved under 2% tensile strain.

## 4. Conclusions

In summary, based on density functional theory calculations, we have systematically explored the electronic structure and optical properties towards photocatalytic water splitting as well as the photovoltaic applications for Sc_2_CF_2_/Sc_2_CCl_2_, Sc_2_CF_2_/Sc_2_CBr_2_, and Sc_2_CCl_2_/Sc_2_CBr_2_ vdW heterostructures. AIMD simulation and phonon spectrum results show that the Sc_2_CF_2_/Sc_2_CCl_2_, Sc_2_CF_2_/Sc_2_CBr_2_, and Sc_2_CCl_2_/Sc_2_CBr_2_ heterostructures are thermally and dynamically stable. Sc_2_CF_2_/Sc_2_CCl_2_, Sc_2_CF_2_/Sc_2_CBr_2_, and Sc_2_CCl_2_/Sc_2_CBr_2_ heterostructures exhibit type-II band alignments with the CBM and VBM located in different monolayers. By further analyzing the band alignment and charge carrier transfer processes, the Sc_2_CF_2_/Sc_2_CCl_2_ and Sc_2_CF_2_/Sc_2_CBr_2_ heterostructures exhibit a direct Z-scheme photocatalyst. These properties can effectively separate the photogenerated carriers, making them suitable for photocatalytic water splitting. Remarkably, a PCE of 20.78% can be achieved for the Sc_2_CCl_2_/Sc_2_CBr_2_ heterostructure, which is higher than that of many other reported heterostructures. In addition, all the heterostructures exhibit excellent optical absorption coefficients in both the visible and UV regions, reaching the order of 10^5^ cm^−1^. This theoretical work demonstrates that the Sc_2_CX_2_/Sc_2_CY_2_ (X, Y = F, Cl, Br) heterostructures are promising candidates for applications in photocatalytic and photovoltaic devices.

## Figures and Tables

**Table 1:** The lattice constants, layer spacing (*d*), and binding energy (*E_b_*) of three possible stackings in Sc_2_CF_2_/Sc_2_CCl_2_, Sc_2_CF_2_/Sc_2_CBr_2_, and Sc_2_CCl_2_/Sc_2_CBr_2_ heterostructures.

System	Configuration	Lattice Constants *a* (Å)	*d* (Å)	*E_b_* (meV*Å^−2^)
Sc_2_CF_2_	-	3.235	-	-
Sc_2_CCl_2_	-	3.422	-	-
Sc_2_CBr_2_	-	3.499	-	-
Sc_2_CF_2_/Sc_2_CCl_2_	A	3.321	2.74	−35.06
	B	3.320	3.13	−35.67
	C	3.321	2.69	−34.77
Sc_2_CF_2_/Sc_2_CBr_2_	A	3.356	2.84	−27.74
	B	3.356	3.24	−28.53
	C	3.357	2.81	−27.35
Sc_2_CCl_2_/Sc_2_CBr_2_	A	3.458	3.23	−19.36
	B	3.458	3.62	−19.96
	C	3.459	3.20	−18.90

## Data Availability

Data are contained within the article and Supplementary Materials.

## Supplementary Materials

The following supporting information can be downloaded at https://www.mdpi.com/article/10.3390/molecules29122898/s1, Table S1: The structure coordinate information (POSCAR) of the Sc_2_CF_2_/Sc_2_CCl_2_ heterostructure with three different stacking configurations. The POSCAR of Sc_2_CF_2_/Sc_2_CBr_2_ and Sc_2_CCl_2_/Sc_2_CBr_2_ heterostructures are the same as those of the Sc_2_CF_2_/Sc_2_CCl_2_ heterostructure; Figure S1: (a–c) AIMD fluctuations of the total energy for the Sc_2_CF_2_/Sc_2_CCl_2_, Sc_2_CF_2_/Sc_2_CBr_2_, and Sc_2_CCl_2_/Sc_2_CBr_2_ heterostructures at 300 K with 6ps. The insets are top and side views of the final structures in the AIMD simulation; Figure S2: (a–c) Phonon dispersion structures of the Sc_2_CF_2_/Sc_2_CCl_2_, Sc_2_CF_2_/Sc_2_CBr_2_, and Sc_2_CCl_2_/Sc_2_CBr_2_ heterostructures; Figure S3: Electrostatic potential for (a) Sc_2_CF_2_, (b) Sc_2_CCl_2_, and (c) Sc_2_CBr_2_ monolayers; Figure S4: Relation between band gap of the Sc_2_CF_2_/Sc_2_CCl_2_ heterostructure and biaxial strain; Figure S5: The projected band structures of the Sc_2_CF_2_/Sc_2_CBr_2_ heterostructure under different vertical strains; Figure S6: Energy bands of the Sc_2_CCl_2_/Sc_2_CBr_2_ heterostructure under different strains; Figure S7: The PCE of the Sc_2_CCl_2_/Sc_2_CBr_2_ heterostructure with different strains.
