# Supplementary material for: First-Principles Study on Janus-Structured Sc2CX2/Sc2CY2 (X, Y = F, Cl, Br) Heterostructures for Solar Energy Conversion
Source: Molecules. 2024 Jun 18;29(12):2898. doi: 10.3390/molecules29122898 (PMC11206758; doi:10.3390/molecules29122898)
Supplement: Supplementary file 1 [file molecules-29-02898-s001.zip › molecules-3038072-supplementary.pdf]

Electronic Supplementary Material for

First-principles study on Janus structured Sc_2CX_2/Sc_2CY_2 (X, Y = F, Cl, Br) Heterostructures for Solar Energy Conversion

Xin He [1,2], Yanan Wu [1], Jia Luo [1], Xianglin Dai [1], Jun Song [1,2] and Yong Tang [1,2,*]

[1] School of Energy Engineering, Huanghuai University, Zhumadian 463000, China; 20202086@huanghuai.edu.cn (X.H.); wuyanan@huanghuai.edu.cn (Y.W.); 10505182024@163.com (J.L.); 20202131@huanghuai.edu.cu (X.D.); songjunaa@163.com (J.S.)

[2] Henan Key Laboratory of Smart Lighting, Huanghuai University, Zhumadian 463000, China

* Correspondence: 20202127@huanghuai.edu.cn

Table S1. The structure coordinate information (POSCAR) of the Sc_2CF_2/Sc_2CCl_2 heterostructure with three different stacking configurations. The POSCAR of Sc_2CF_2/Sc_2CBr_2 and Sc_2CCl_2/Sc_2CBr_2 heterostructures are the same as those of the Sc_2CF_2/Sc_2CCl_2 heterostructure.

A		
1.0		
3.2667000294	0.0000000000	0.0000000000
-1.6333500147	2.8290452120	0.0000000000
0.0000000000	0.0000000000	39.9822006226
Sc F Cl C		
4 2 2 2		
Direct		
0.333333343	0.666666687	0.637989998
0.333333343	0.666666687	0.442160010
0.666666687	0.333333343	0.574590027
0.666666687	0.333333343	0.378760010
0.333333343	0.666666687	0.545889974
0.666666687	0.333333343	0.666666687
0.333333343	0.666666687	0.350059986
0.666666687	0.333333343	0.470860004
0.000000000	0.000000000	0.606289983
0.000000000	0.000000000	0.410459995
B		
1.0		
3.2667000294	0.0000000000	0.0000000000
-1.6333500147	2.8290452120	0.0000000000
0.0000000000	0.0000000000	39.9822006226
Sc F Cl C		
4 2 2 2		
Direct		
0.666666687	0.333333343	0.637989998
0.666666687	0.333333343	0.378760010
0.333333343	0.666666687	0.442160010
0.000000000	0.000000000	0.574590027
0.666666687	0.333333343	0.545889974
0.000000000	0.000000000	0.666666687
0.666666687	0.333333343	0.470860004
0.333333343	0.666666687	0.350059986
0.333333343	0.666666687	0.606289983
0.000000000	0.000000000	0.410459995
C		
1.0		
3.2667000294	0.0000000000	0.0000000000
-1.6333500147	2.8290452120	0.0000000000
0.0000000000	0.0000000000	39.9822006226
Sc F Cl C		
4 2 2 2		
Direct		
0.000000000	0.000000000	0.637989998
0.333333343	0.666666687	0.442160010
0.333333343	0.666666687	0.574590027
0.666666687	0.333333343	0.378760010
0.000000000	0.000000000	0.545889974
0.333333343	0.666666687	0.666666687
0.333333343	0.666666687	0.350059986
0.666666687	0.333333343	0.470860004
0.666666687	0.333333343	0.606289983
0.000000000	0.000000000	0.410459995

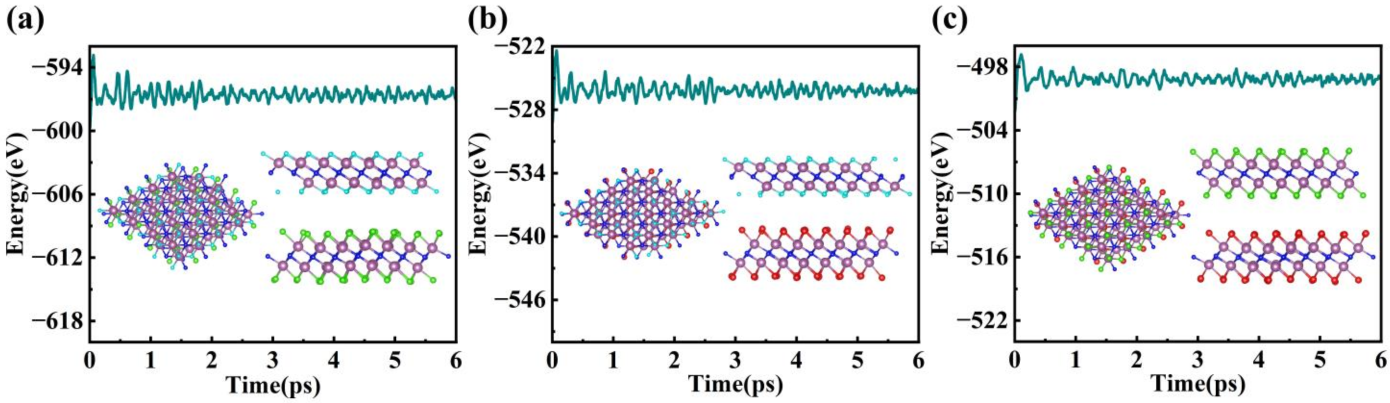

Figure S1. (a-c) AIMD fluctuations of the total energy for the Sc_2CF_2/Sc_2CCl_2, Sc_2CF_2/Sc_2CBr_2, and Sc_2CCl_2/Sc_2CBr_2 heterostructures at 300 K with 6ps. The insets are top and side views of the final structures in the AIMD simulation.

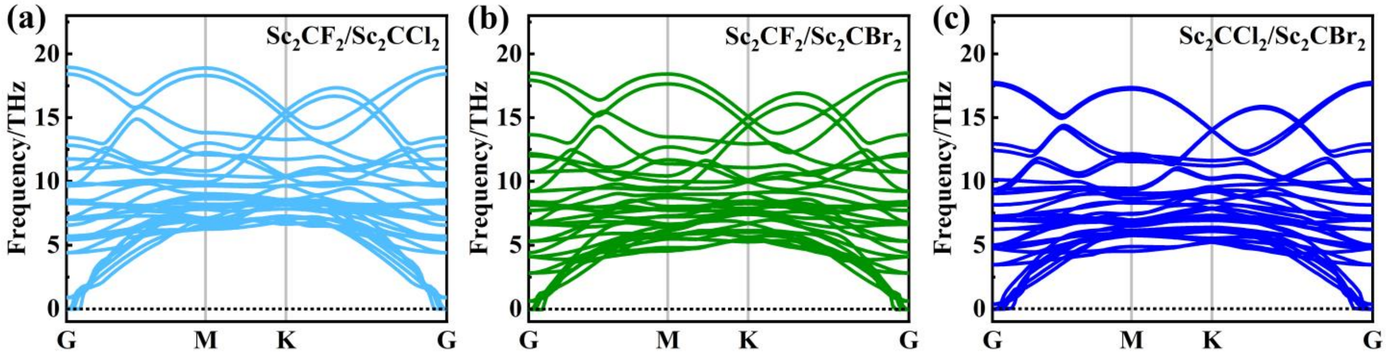

Figure S2. (a-c) Phonon dispersion structures of the Sc_2CF_2/Sc_2CCl_2, Sc_2CF_2/Sc_2CBr_2, and Sc_2CCl_2/Sc_2CBr_2 heterostructures.

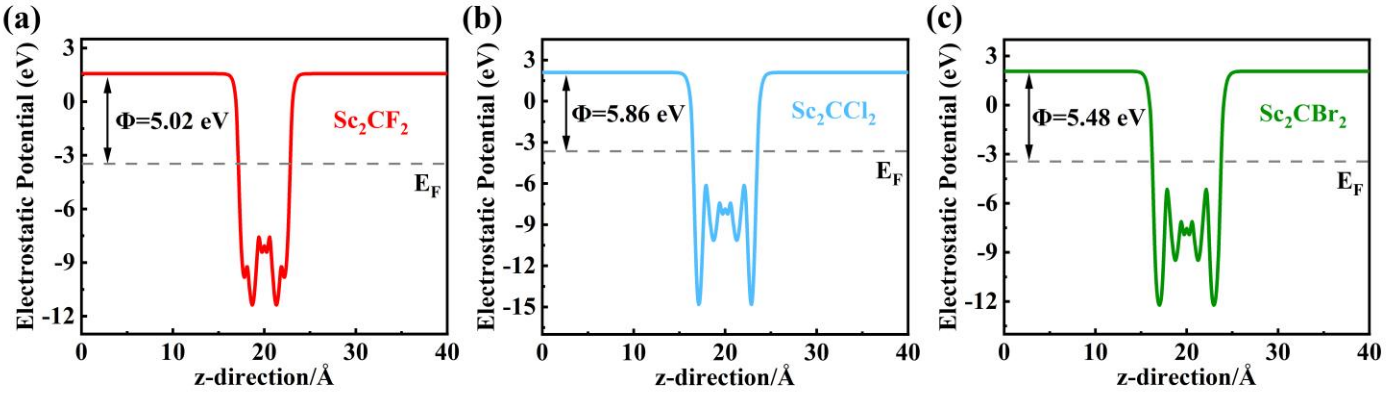

Figure S3. Electrostatic potential for (a) Sc_2CF_2, (b) Sc_2CCl_2, and (c) Sc_2CBr_2 monolayers.

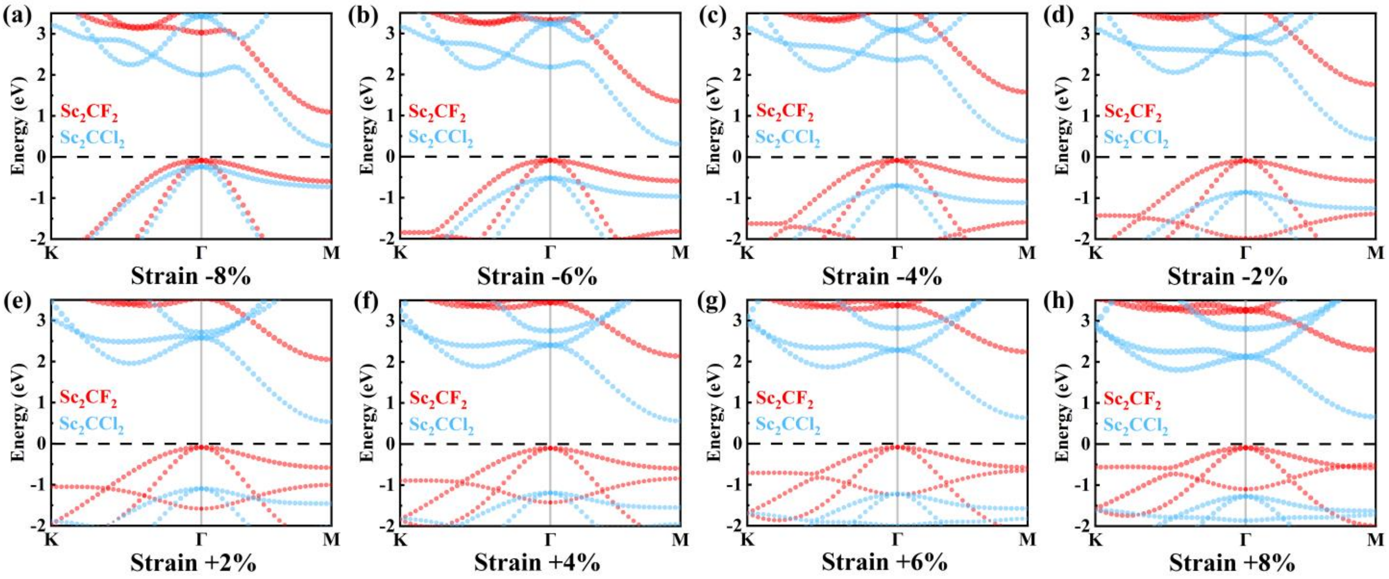

Figure S4. Relation between band gap of the Sc_2CF_2/Sc_2CCl_2 heterostructure and biaxial strain.

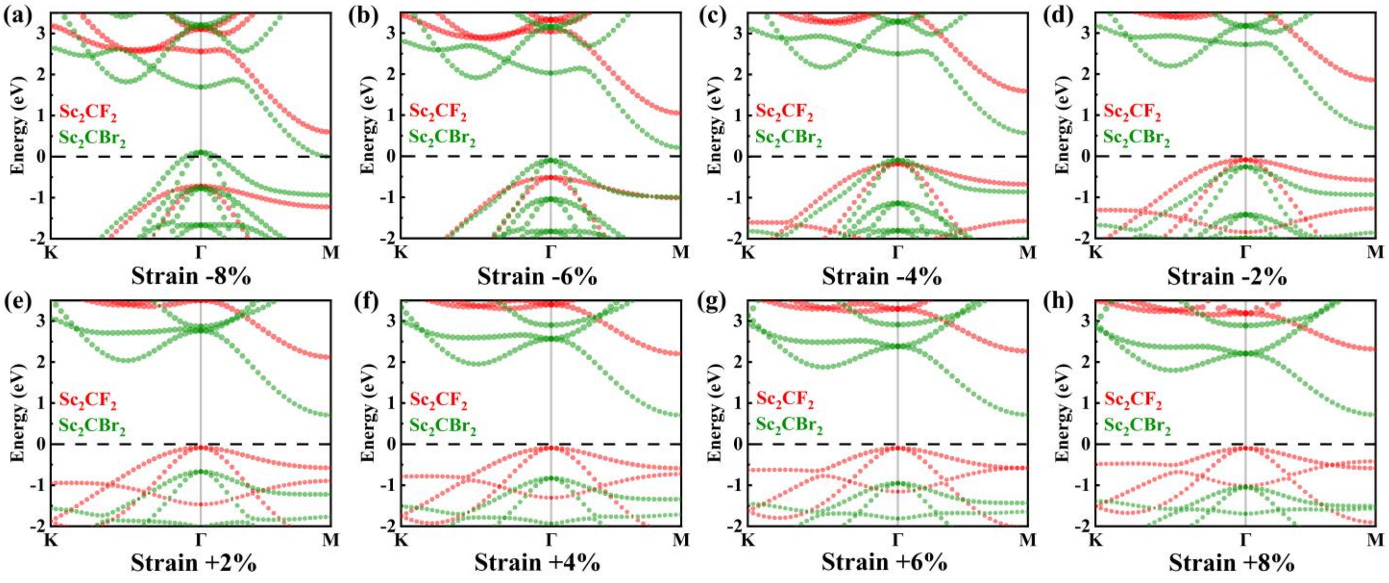

Figure S5. The projected band structures of the Sc_2CF_2/Sc_2CBr_2 heterostructure under different vertical strains.

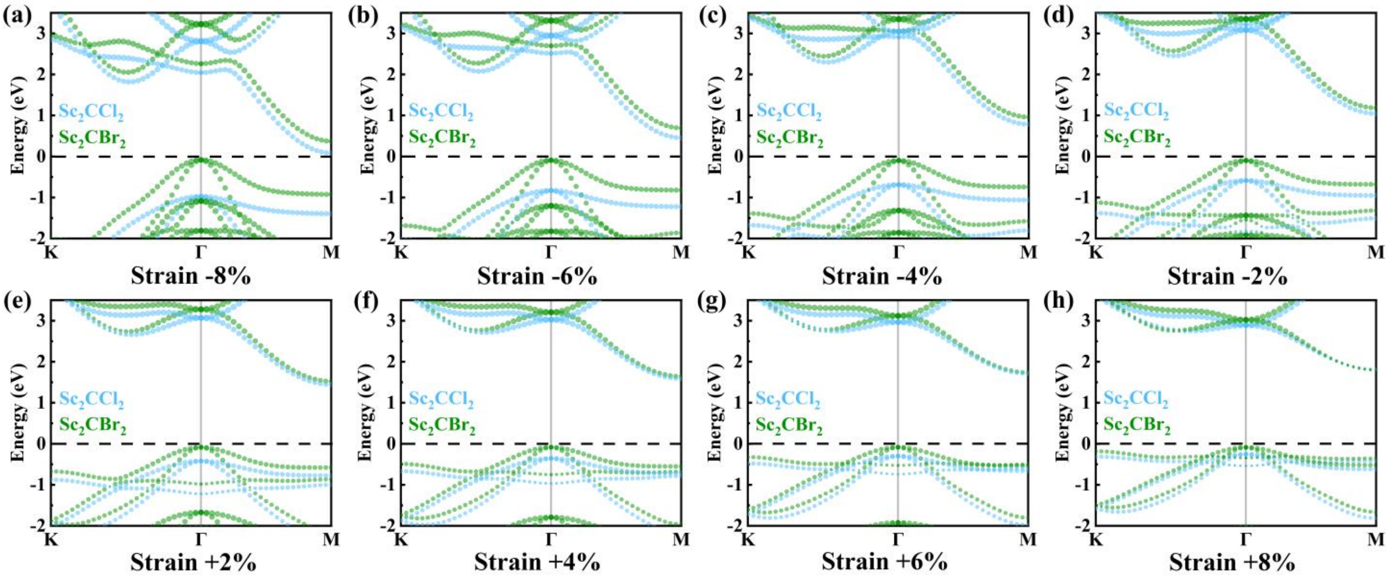

Figure S6. Energy bands of the Sc_2CCl_2/Sc_2CBr_2 heterostructure under different strains.

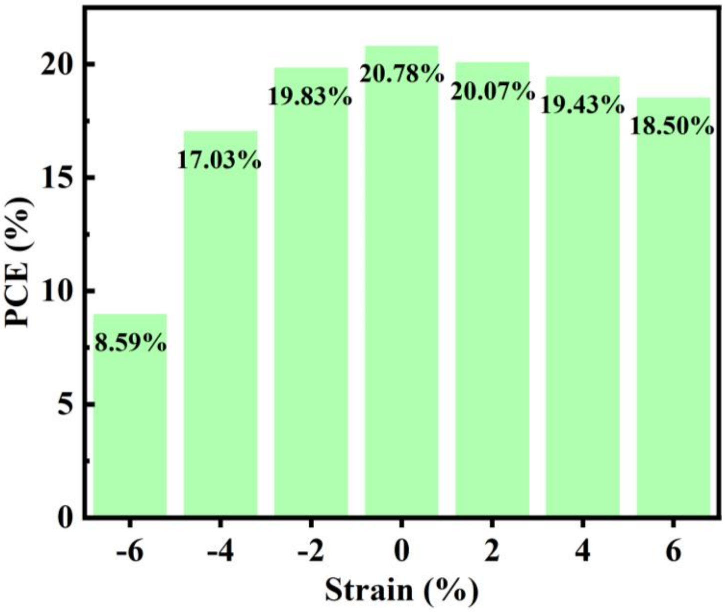

Figure S7. The PCE of the Sc_2CCl_2/Sc_2CBr_2 heterostructure with different strains.
